# Mechanism of *Rhodiola rosea*–*Euonymus alatus* drug pair against rheumatoid arthritis: Network pharmacology and experimental validation

**DOI:** 10.1002/iid3.1127

**Published:** 2023-12-26

**Authors:** Qiu‐han Zheng, Lian‐yun Du, Ying Zhao, Zhong Zhang, Song‐lan Piao, Ying‐hang Wang, Zhi Pan

**Affiliations:** ^1^ Jilin Ginseng Academy Changchun University of Chinese Medicine Changchun People's Republic of China; ^2^ Clinical College of Integrated Chinese and Western Medicine Changchun University of Chinese Medicine Changchun People's Republic of China; ^3^ Clinical Medical School Changchun University of Chinese Medicine Changchun People's Republic of China; ^4^ The Affiliated Hospital of Changchun University of Chinese Medicine Changchun People's Republic of China

**Keywords:** *Euonymus alatus*, fibroblast‐like synovial cells in human rheumatoid arthritis, mechanism of action, network pharmacology, rheumatoid arthritis, *Rhodiola rosea*

## Abstract

**Purpose:**

The present study aimed to explore the potential components and mechanisms of *Rhodiola rosea*–*Euonymus alatus* drug pair (TY) that ameliorate rheumatoid arthritis (RA).

**Methods:**

The main active components, core targets, and important pathways of TY against RA were predicted by network pharmacology analysis. The binding activity between the main active components and the core targets was verified by the molecular docking technique. Collagen‐induced arthritis (CIA) rat model and tumor necrosis factor (TNF)‐α‐induced fibroblast‐like synovial cells in human RA (HFLS‐RA) model were established, respectively. The core targets were verified by cell counting kit‐8 assay, hematoxylin eosin, enzyme‐linked immunosorbent assay, real‐time polymerase chain reaction, and Western blot analysis, and the therapeutic effect of TY was evaluated.

**Results:**

A total of 18 possible components and 34 core targets were obtained by network pharmacology, among which inflammatory response, phosphatidylinositide 3‐kinases (PI3K)‐AKT and MAPK pathways were involved in the therapeutic effect of TY on RA. The results of molecular docking showed that kaempferol and quercetin had high binding affinity to interleukin (IL)‐1β, IL‐6, matrix metalloproteinase (MMP)9, and TNF‐α. In vivo and in vitro experiments showed that TY dose‐dependently inhibited the proliferation of HFLS‐RA cells induced by TNF‐α, and significantly reduced the paw swelling and arthritis scores in CIA rats. At the same time, TY inhibited the production of inflammatory factors in CIA rat serum and TNF‐α‐induced HFLS‐RA cells. It also decreased the expression of PI3K, phospho‐protein kinase B, MMP1, MMP3, MMP9, and increased the protein and mRNA levels of tissue inhibitors of MMPs (TIMP)1 in synovial tissue.

**Conclusion:**

TY can inhibit the PI3K/AKT signaling pathway and regulate the balance between MMPs and TIMP, thus playing a therapeutic role in RA.

## Graphical abstract

In this study, we adopted a comprehensive approach to uncover the potential components and mechanisms of *Rhodiola rosea*–*Euonymus alatus* drug pair (TY) that ameliorate rheumatoid arthritis (RA) via network pharmacology integrated molecular docking strategy. We also confirmed our findings by in vivo and in vitro experiments, which TY can improve the symptoms of arthritis in rats, alleviate the inflammatory response and angiogenesis, and inhibit the proliferation of ibroblast‐like synovial cells in human RA cells. The underlying mechanism may be related to inhibiting the phosphatidylinositide 3‐kinases/protein kinase B signaling pathway and regulating the balance between matrix metalloproteinases and tissue inhibitors of MMPs.

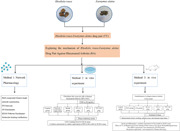

## 1 INTRODUCTION

Rheumatoid arthritis (RA) is a chronic systemic autoimmune disease characterized by synovitis and destructive arthropathy. Chronic inflammation of synovial membrane promotes pannus formation around the joint as the basic pathological change in RA, which in turn erodes articular cartilage, bone, and surrounding tissues, eventually leading to joint injury, deformity, dysfunction, and even disability.^1^ Studies have shown that the worldwide prevalence of RA is 0.5%–1.0%. Compared with normal people, patients with RA are susceptible to major diseases such as infections, which seriously affect their quality of life.^2^ The pathogenesis of RA is complex, involving genetic factors, environmental factors, immune dysregulation, uncontrolled cytokine release, and other aspects. Therefore, it is necessary to find safer and more cost‐effective anti‐inflammatory drugs for RA. Traditional Chinese medicine may provide some new options, such as traditional Chinese medicine active ingredients Quercetin, Caffeic acid, Oxymatrine, Triptolide and so on, which have been proved to have a good therapeutic effect on RA.^3^, ^4^

*Rhodiola rosea*, a perennial herb of *Rhodiola L*. of *Crassulaceae*, also known as “Golden Root” or “Roseroot,” is a valuable medicinal plant that has appeared in many European countries. *R. Rosea* is flat, astringent, and good for moistening the lungs. It tonifies the kidneys, regulates Qi, and nourishes the blood. *Euonymus alatus*, first recorded in Shennong's Herbal Classic, is a winged twig or winged appendage of *E. alatus (Thunb.) Sieb*. *E. alatus*, bitter and cold in nature, belongs to the liver meridian and has the effect of breaking blood and opening the meridians, detoxifying and eliminating swellings, and killing insects. Experimental studies have found that the extract of *R. rosea* alleviates adjuvant arthritis‐associated joint damage by inhibiting NF‐κB and RANK/RANKL/OPG signaling pathways.^5^ *E. alatus* with blood‐breaking and meridian‐unblocking properties effectively alleviated RA caused by blocking meridians and poor circulation of Qi and blood. Although several studies have focused on the anti‐RA effects of *R. rosea* or *E. alatus*, the underlying mechanisms of *R. rosea–E. alatus* drug pair (TY) have not been fully elucidated.

**Figure 1:**
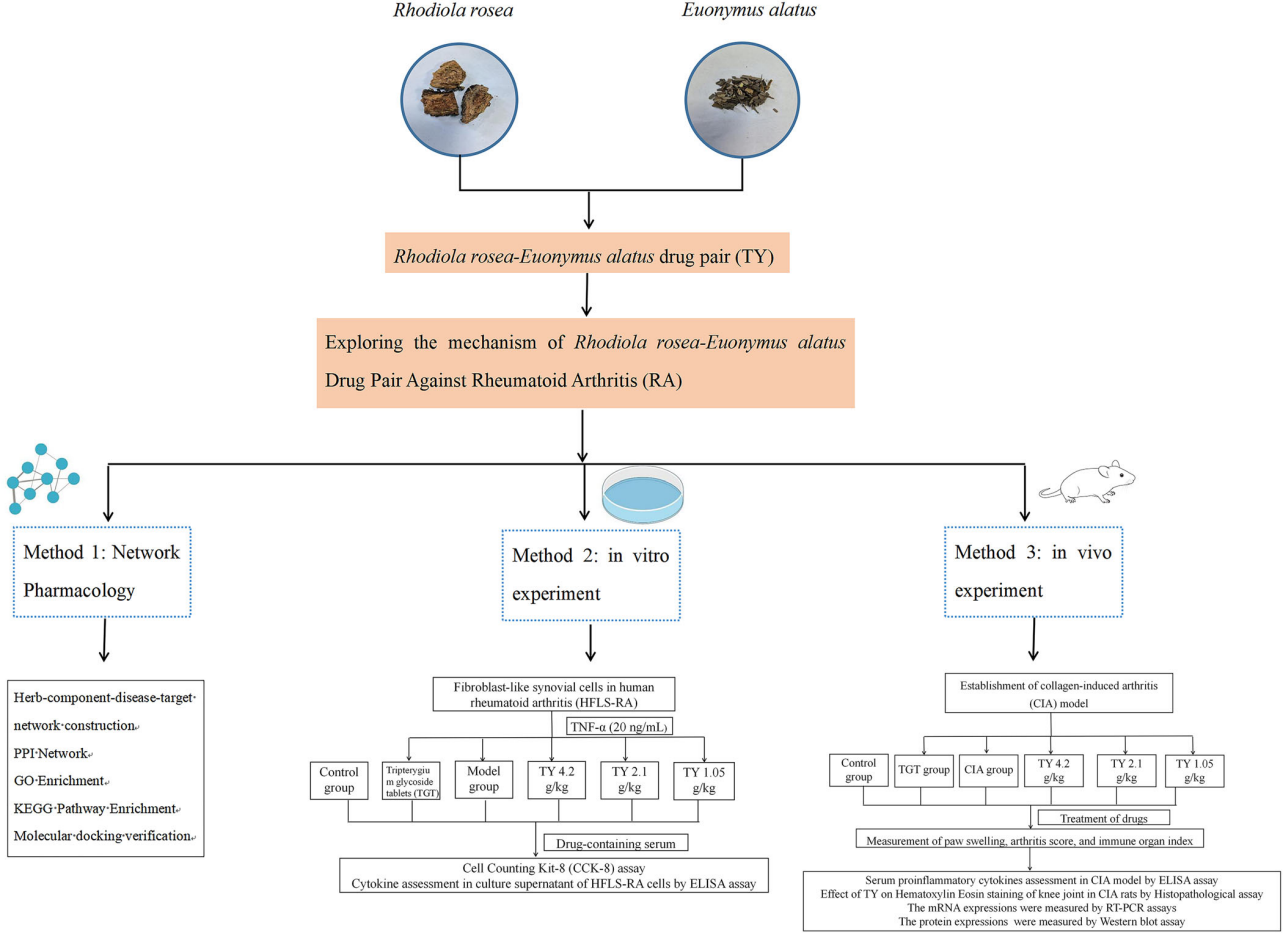
Technical strategy of the current study.

Network pharmacology is a promising method for dissecting complex mechanisms and drug discovery.^6^ Therefore, in this study, network pharmacology was used to identify the therapeutic targets and related pathways of TY against RA. We also confirmed our findings by in vivo and in vitro experiments. Our findings can illuminate the components and mechanisms of TY that can alleviate RA, and provide a theoretical basis for utilizing TY in clinical practice. The technical strategy of the current study is shown in Figure 1.

## 2 MATERIAL AND METHODS

### 2.1 Reagents and materials

*R. rosea* was obtained from Beijing Qiancao Chinese Medicine Pill Company. *E. alatus* was obtained from Anxing Chinese Herbal Medicine CO. Tripterygium glycoside tablets (TGT) were purchased from Forward. Recombinant human tumor necrosis factor (TNF)‐α was obtained from Peprotech. Enzyme‐linked immunosorbent assay (ELISA) kits of human or rat interleukin (IL)‐6, IL‐1β, and TNF‐α were purchased from Enzyme‐linked Biotechnology. Cell counting kit‐8 (CCK‐8) solution, RIPA tissue lysis buffer, and bicinchoninic acid (BCA) protein assay kit were purchased from Beyotime Institute of Biotechnology Co., Ltd. Primary antibodies against phosphatidylinositide 3‐kinases (PI3K), phospho‐protein kinase B (p‐AKT), matrix metalloproteinase (MMP)1, MMP3, MMP9, tissue inhibitors of MMPs (TIMP)1, and glyceraldehyde‐3‐phosphate dehydrogenase (GAPDH) were purchased from ABclonal. Goat antirabbit IgG (H & L)‐HRP secondary antibodies were purchased from ABclonal. The RNA simple total RNA kit was purchased from TIANGEN Biotech Co., Ltd. The Bioteke super RT kit was purchased from Biotake Corporation Co., Ltd., and the 2 × Hieff robust PCR master mix was purchased from Yeasen Biotechnology Co., Ltd. The primers of MMP1, MMP3, MMP9, TIMP1, and β‐actin were produced by Shanghai Sangon Biological and Technological Company. Bovine type II collagen was purchased from Chondrex, Inc. Freund's complete adjuvant (CFA) was purchased from Sigma‐Aldrich. Hematoxylin eosin (H&E), neutral gum were supplied by Beijing Solarbio Science & Technology Co., Ltd.

RT‐6100 microplate reader (Rayto). 165‐8000 electrophoresis apparatus, 170‐3930 transfer membrane apparatus (Bio‐Rad). FL1000 intelligent imaging system (Thermo Fisher Scientific). ETC811 gene amplification instrument (Eastwin). SK‐O180‐E horizontal shaker (DRAGONLAB).

### 2.2 Preparation of TY decoction

According to the Chinese Pharmacopoeia 2020 edition,^7^ two times the dosage of *R. rosea* and *E. alatus* was set as the clinical dosage. Then, according to the equivalent dose ratio index table of human and animal and body surface area listed in Pharmacological Experimental Methodology, the equivalent dose for rats was calculated to be 2.1 g/kg, which was set as the medium dose group. The low dose was 1.05 g/kg and the high dose was 4.2 g/kg. *R. rosea* and *E. alatus* were weighed in a 1:1 ratio and soaked in distilled water for 1 h. Then, boiled in 10‐fold amounts of distilled water twice (1.5 h each time). Filtered solutions were mixed. The decoction was concentrated to the concentration of water decoction, which was 0.105, 0.21, and 0.42 g/mL, respectively and stored at 4°C for later use.

### 2.3 Network pharmacology‐based analysis of TY against RA

#### 2.3.1 Screening of effective components and targets of TY

The main effective components of the *E. alatus* were collected from the TCMSP (https://tcmspw.com/tcmsp.php).^8^ The chemical structure and SMILES of *R. rosea* were obtained from the PubChem website (https://pubchem.ncbi.nlm.nih.gov/compound/442428).^9^ Target prediction of *R. rosea* was conducted using the SwissTargetPrediction database (http://www.swisstargetpredict-ion.ch/).^10^ According to the absorption, distribution, metabolism, and excretion characteristics of the drugs in the body, all components of TY were screened in the following conditions: oral bioavailability ≥30% and drug‐like property ≥0.18.^11^, ^12^ Then, the target proteins were converted into genes via UniProt (http://www.UniProt.org/).^13^ Finally, the genes were combined, and duplicated items were removed to obtain the active ingredients and targets of TY.

#### 2.3.2 Screening of disease‐related targets for RA

RA‐related disease targets were screened from the DisGeNET (http://www.DisGeNET.org/home/), DrugBank (https://www.drugbank.ca), Therapeutic Target database (http://db.idrblab.net/ttd), and GeneCards (https://www.genecards.org/) using the keyword “Rheumatoid Arthritis.”^14^, ^15^, ^16^, ^17^ Data from the four databases were combined, duplicates were removed and imported into the UniProt database to standardize the targets. Finally, a database of RA‐related targets was obtained.

#### 2.3.3 Obtaining potential targets for TY and RA

Active ingredients of TY and RA‐related disease targets were intersected to obtain potential targets for TY in RA. In addition, active ingredients of TY and RA‐related targets were imported into VENNY2.1.0 (https://bioinfogp.cnb.csic.es/tools/venny/) for visualization. The intersection targets were considered potential targets of TY in RA. The intersection targets, active ingredients of TY, Chinese medicine names, and disease names were imported into Cytoscape 3.7.0 software to construct a herbal‐component‐disease‐target network and analyze the topological parameters. This network diagram presents their interrelationships and identifies the core components of TY against RA.

#### 2.3.4 Construction of protein–protein interaction (PPI) network and screening of core targets

The intersection targets were imported into the STRING (https://www.string-db.org/) database to obtain a PPI network diagram.^18^ The organism was set as *Homo sapiens*. The mode was selected as multiple proteins. The TSV‐format file was downloaded from the STRING database and imported into Cytoscape 3.7.0. Using CytoNCA plug‐in, target proteins were calculated, and filtered separately according to the betweenness centrality (BC), closeness centrality (CC), eigenvector centrality (EC), and degree centrality (DC).

#### 2.3.5 GO and KEGG pathway enrichment analyses

The intersection targets were imported into the Matescape (http://metascape.org/gp/index.html) database and the species was limited to *H. sapiens*.^19^ Gene ontology (GO) and kyoto encyclopedia of genes and genomes (KEGG) pathway enrichment analyses were conducted using the Metascape database. The bar chart and histogram were visualized by the bioinformatics website (http://www.bioinformatics.com.cn/).

#### 2.3.6 Molecular docking verification

We downloaded the 2D structures of active ingredients from the Pub Chem database in SDF format. The Chem3D software was used to optimize the structures and obtain the mol2 format files of the 3D structures. The corresponding PDB ID of core targets was found in the PBD (http://www1.rcsb.org/) database and saved as the PDB format. The PyMOL software (Version 2.5, https://pymol.org/2/) was used to remove water molecules and small‐molecule ligands from receptors. Finally, molecular docking was performed by the AutoDock Vina software (The Scripps Research Institute, version 1.2.0) to calculate the binding score of the main active components to core targets, and 2D and 3D visualization analysis was performed using LigPlot^+^ versions 2.2.8 and PyMOL2.5 software.

### 2.4 In vitro experimental study of TY against RA

#### 2.4.1 Preparation of drug‐containing serum

Specific pathogen‐free healthy male Wistar rats (200 ± 20 g) were provided by Changchun Yisi Experimental Animal Technology Co. (production license: SCXK, 2020‐0002). All animals were housed in a temperature‐controlled room (23 ± 2°C), with 50%–65% relative humidity and 12 h:12 h dark and light cycles. Animals had free access to food and water. Before the experiment, 1 week was spent on adaptive feeding.

In total, 36 male Wistar rats were randomly divided into six groups (*n* = 6) according to the random number table: the control group, collagen‐induced arthritis (CIA) model group, low‐dose TY group (1.05 g/kg), medium‐dose TY group (2.1 g/kg), high‐dose TY group (4.2 g/kg), and positive group (TGT, 9.45 mg/kg). TGT is routinely used to treat RA in the long term. The drug was administered 10 mL/kg/day via oral administration for 1 week. The control and CIA model groups were given an equal volume of distilled water. Two hours after the last dose, blood samples were collected from the abdominal aorta and kept at room temperature for 2 h. Then, blood samples were centrifuged at 3000 rpm for 10 min, the supernatant was inactivated in water bath 56°C for 30 min and the bacteria were removed by a 0.22 µm filtration membrane. Finally, the supernatant was kept at −20°C.

#### 2.4.2 Cell culture, cell grouping, and intervention

Fibroblast‐like synovial cells in human RA (HFLS‐RA) were purchased from Shanghai Jin Yuan Biological Co. and cultured in the minimum essential medium (MEM) with 10% fetal bovine serum (Hyclone) and 1% penicillin/streptomycin (Hyclone). Cells were incubated in humidified conditions with 5% CO_2_ at 37°C. When the cell density reached 80%–90%, the cells were detached by 0.25% trypsin, passaged, and used for subsequent experimental studies.

The cells were divided into the blank group (10% blank group drug‐containing serum), model group (20 ng/mL TNF‐α + 10% model group drug‐containing serum), positive group (20 ng/mL TNF‐α + 10% positive group drug‐containing serum), and low‐, medium‐, and high‐dose TY group (20 ng/mL TNF‐α + 10% TY at low‐, medium‐, and high‐dose group drug‐containing serum).

#### 2.4.3 CCK‐8 assay

The effect of TY on HFLS‐RA cell viability was measured by the CCK‐8 assay. The HFLS‐RA cells (5 × 10^4^ cells/well) were seeded in 96‐well plates with a total volume of 100 μL/well and cultured overnight in the CO_2_ incubator. The culture medium was discarded, 100 μL of MEM was added to the blank group, and 100 μL of 20 ng/mL TNF‐α was added to other groups. After 1 h, the culture media was discarded, the drug‐containing serum was added, and the cells were cultured in the CO_2_ incubator for 24 h. Then, 10 μL of CCK‐8 solution was added to each well and further incubated for 2 h. The optical density of each well was measured at 450 nm wavelength with a microplate reader and the inhibition rate was calculated.

#### 2.4.4 Cytokine assessment in the culture supernatant of HFLS‐RA cells by ELISA assay

The effect of TY on HFLS‐RA cell cytokine production was measured by ELISA assay. HFLS‐RA cells (1 × 10^5^ cells/well) were seeded in 6‐well plates with a total volume of 1 mL/well and cultured overnight in the CO_2_ incubator. Cell stimulation and drug delivery were done according to the above steps. Cell supernatants were collected after centrifugation and IL‐1β, IL‐6, and TNF‐α levels were analyzed with an ELISA kit.

### 2.5 In vivo experimental study of TY against RA

#### 2.5.1 Establishment of the CIA model and treatment

The CIA rat model was used in this work, because it can well simulate the pathological characteristics of human RA, such as decreased muscle weight, increased fatigue, and weakness.^20^, ^21^ In total, 60 male Wistar rats were randomly assigned to the groups according to the previous method. Bovine collagen type II was dissolved in 0.05 M acetic acid to obtain 2 mg/mL concentration, and stood overnight under 4°C. Then, bovine collagen type II solution was mixed with complete CFA at a 1:1 ratio to obtain a stable and homogeneous emulsion. For initial immunization, 0.15 mL of the emulsion was intradermally injected into the right metatarsal footpad except for the control group. Seven days later, 0.1 mL of emulsion was administered at the same sites to boost immunity.^22^ Two weeks later, the positive group was treated with TGT (9.45 mg/kg) and TY groups were given the corresponding doses of TY (1.05, 2.1, and 4.2 g/kg/day) for 4 weeks. The control and CIA model groups were given an equal volume of distilled water. During housing, animals were monitored twice daily for health status. No adverse events were observed.

#### 2.5.2 Measurement of paw swelling, arthritis score, and immune organ index

During treatment, the body weight of rats and the perimeter of the knee joint were measured every 7 days. Meanwhile, the severity of arthritis was scored by two researchers in a blind manner. Each paw was scored on a four‐grade scale (1–4), and the maximum grade was 16. The semiquantitative scoring system was defined as follows: 0 = no erythema and swelling of the paw; 1 = slight swelling or redness in toe; 2 = swelling and redness of the paws; 3 = severe swelling and redness in the ankles and feet; 4 = serious swelling and redness in the whole leg and all digits and no bearing of its own weight.^23^ Then, the arthritis index and swelling rate were calculated to reflect the severity of arthritis. Eventually, the thymus and spleen of the rats were harvested, rinsed in normal saline, dried on filter paper, weighed, and the immune organ index was calculated according to the following formula: immune organ index = absolute organ weight/body weight.

#### 2.5.3 Histopathological analysis

On the 42nd day of the experiment, the rats were fasted for 12 h and then anesthetized with the intraperitoneal injection of 20% ethyl carbamate at a dose of 0.7 mL/100 g. The knee joints of the rats were carefully excised. Afterward, the knee joints of rats were fixed in 4% paraformaldehyde for 48 h, and then decalcified in decalcification solution. After dehydration, transparency, embedding, slicing (5 μm), drying, dewaxing, and other steps, H&E staining was done, and the pathological changes of knee joints were observed and evaluated using an inverted fluorescence microscope.

#### 2.5.4 Proinflammatory cytokine detection by ELISA assay

Blood samples were collected from the abdominal aorta and kept at room temperature for 2 h. Then, Blood samples were centrifuged at 3000 rpm for 10 min. The serum levels of IL‐1β, IL‐6, and TNF‐α were determined by ELISA kits according to the instructions, with the absorption intensity value determined at 450 nm using a microplate reader.

#### 2.5.5 Real‐time polymerase chain reaction (RT‐PCR)

The mRNA expression of MMP1, MMP3, MMP9, and TIMP1 in the synovial tissue was measured by RT‐PCR. Synovial tissues were ground using a tissue grinder, and then total RNA was extracted using the total RNA kit. A nucleic acid quantifier was used to quantify mRNA, and 1 µg of total RNA was used for reverse transcription to synthesize cDNA. Subsequently, 2 × Hieff PCR Master Mix, template, primers, and water were mixed into a 50 μL system. The template was then amplified by RT‐PCR to obtain the Ct value of each sample. The relative expression of each target gene was quantified and normalized by the $2^{‐∆∆C_{t}}$ method. The primers are listed in Table 1. All experiments were done in triplicate.

**Table 1:** The sequence of primers for RT‐PCR.

Gene name	Description	Primer sequences
MMP1	Forward	GGTCTCTGAGGGTCAAGCAG
	Reverse	AGTTCATGAGCTGCAACACG
MMP3	Forward	AGTCTTCCAATCCTACTGTTGCT
	Reverse	TCCCCGTCACCTCCAATCC
MMP9	Forward	TGTACCGCTATGGTTACACTCG
	Reverse	GGCAGGGACAGTTGCTTCT
TIMP1	Forward	ACCACCTTATACCAGCGTTATGA
	Reverse	GGTGTAGACGAACCGGATGTC
β‐actin	Forward	GTCGTACCACTGGCATTGTG
	Reverse	TCTCAGCTGTGGTGGTGAAG

Abbreviations: MMP, matrix metalloproteinase; RT‐PCR, real‐time polymerase chain reaction; TIMP, tissue inhibitors of MMPs.

#### 2.5.6 Western blot analysis

Western blot analysis was used to determine the expression of PI3K, p‐AKT, MMP1, MMP3, MMP9, TIMP1, and GAPDH in the synovial tissue of rats. Protein was extracted from the synovial tissue using RIPA lysis buffer with 1% PMSF. The protein concentration was quantified using the BCA protein assay kit. Equal amounts of protein samples were subjected to 10% sodium dodecyl sulfate polyacrylamide gel electrophoresis and transferred to polyvinylidene fluoride membranes. The membranes were blocked with 5% skim milk for 1 h on a horizontal shaker at room temperature and incubated at 4°C overnight with primary antibodies against PI3K (1:500), p‐AKT (1:500), MMP1 (1:500), MMP3 (1:500), MMP9 (1:500), TIMP1 (1:500), and GAPDH (1:1000), respectively. After washing with tris‐buffered saline tween, the membranes were incubated for 1 h on a horizontal shaker at room temperature with goat anti‐rabbit IgG (H & L)‐HRP secondary antibody. Finally, the protein bands were detected using the enhanced chemiluminescence detection reagent. Images were quantified using Image J software and normalized to GAPDH. All experiments were done in triplicate.

### 2.6 Statistical analysis

The statistical analyses were performed using GraphPad Prism 9.5 (GraphPad Software. Data from these experiments are expressed as mean ± standard deviation. Differences between groups were evaluated by one‐way analysis of variance with Tukey's multiple comparisons test, and *p* < .05 was considered statistically significant.

## 3 RESULTS

### 3.1 Results of network pharmacology‐based analysis of TY against RA

#### 3.1.1 Obtaining active components and potential targets of TY

In total, 26 active constituents of *R. rosea* and 24 active constituents of *E. alatus* were obtained. Based on the screening condition, 18 compounds in TY were collected, among which 11 belonged to *R. rosea*, 8 belonged to *E. alatus*, and one was a shared component (Table 2). In total, 216 targets of effective components of *R. rosea* and 251 targets of *E. alatus* were obtained. After integration with the UniProt database and deleting duplicate targets, 326 targets were obtained.

**Table 2:** Main active constituents of TY.

Sources	Main active ingredient	Sign
*Rhodiola rosea*	3‐Octanol	HJT1
	Hexanal	HJT2
	Salidroside	HJT3
	Linalool oxide	HJT4
	Myrtanol	HJT5
	Myrtenol	HJT6
	Octanal	HJT7
	Octanoic acid	HJT8
	1‐Octanol	HJT9
	3‐Methyl‐2‐butenal	HJT10
Shared	Kaempferol	A1
*Euonymus alatus*	(2R)−5,7‐dihydroxy‐2‐(4‐hydroxyphenyl)chroman‐4‐one	GJY1
	ZINC04073977	GJY2
	24‐Ethylcholest‐4‐en‐3‐one	GJY3
	Beta‐sitosterol	GJY4
	Sitosterol	GJY5
	5,7‐Dihydroxy‐2‐(3‐hydroxy‐4‐methoxyphenyl)chroman‐4‐one	GJY6
	Quercetin	GJY7

Abbreviation: TY, *Rhodiola rosea*–*Euonymus alatus* drug pair.

#### 3.1.2 Acquisition of RA‐related targets

**Figure 2:**
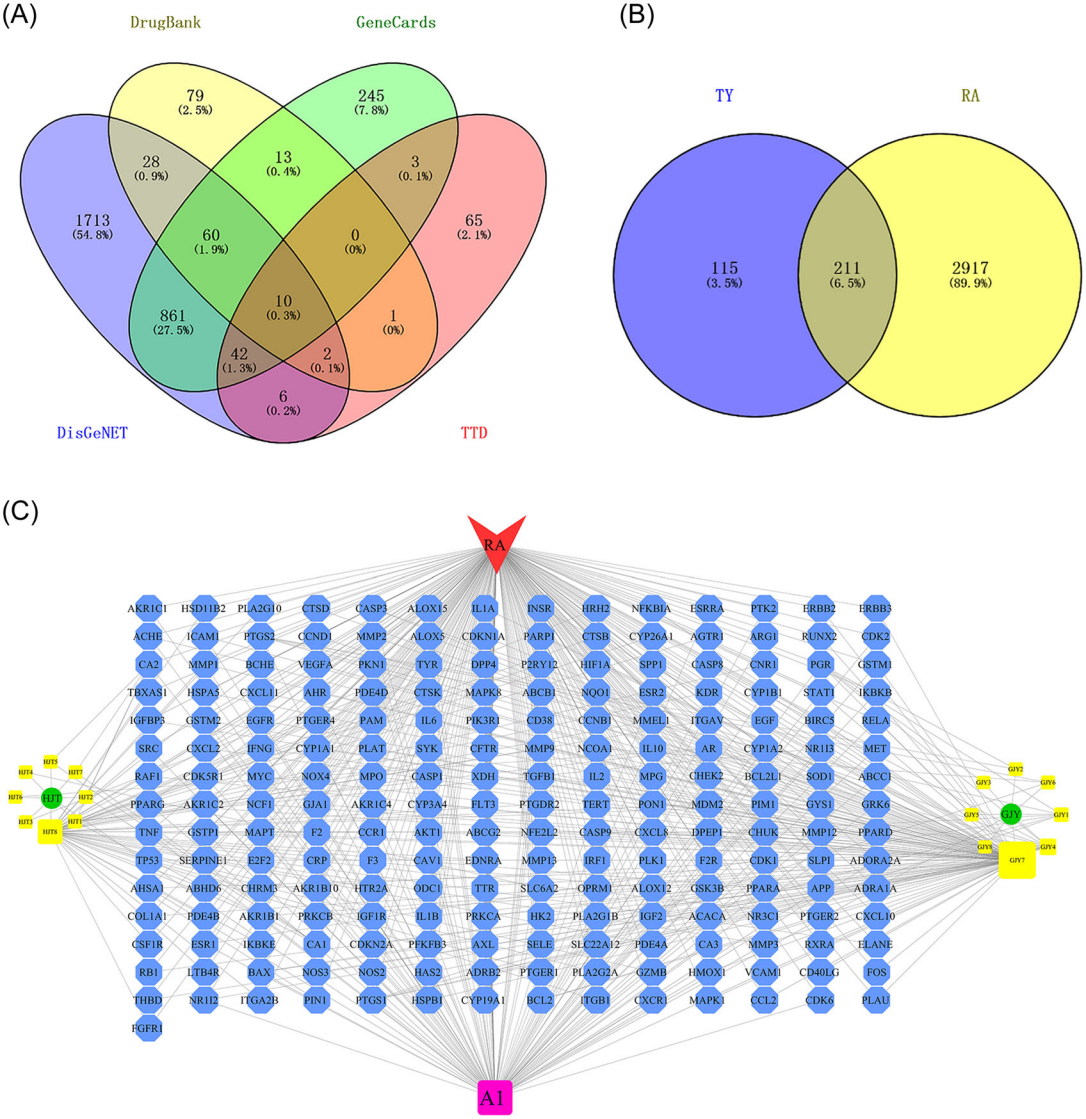
Targets involved in therapeutic effects of *Rhodiola rosea*–*Euonymus alatus* drug pair (TY) on rheumatoid arthritis (RA). (A) Venn diagram of RA targets. (B) Venn diagram of TY targets and RA targets showing that 211 targets overlapped between TY and RA. (C) Herb‐active component‐disease‐target network. The red triangle represents RA, the blue hexagon represents the potential target of TY in RA, the green circle represents *R. rosea* and *E. alatus*, the yellow quadrilateral represents the active constituents of TY, and the purple quadrilateral represents the shared active constituents of TY.

We collected 2723 RA‐related targets from the DisGeNET database; 1234 RA‐related targets from the GeneCards database; 594 RA‐related targets from the DrugBank; and 147 RA‐related targets from the therapeutic target database (TTD). After deleting duplicate targets, 3128 pathogenic genes related to RA were obtained from the aforementioned databases (Figure 2A).

#### 3.1.3 Intersectional targets of TY and RA

The 326 targets of TY and the 3128 RA‐related targets were imported into VENNY2.1.0. Then, 211 intersection targets which were the potential targets of TY in RA were obtained and shown in a Venn diagram (Figure 2B). A herb‐active component‐disease‐target network was visualized using Cytoscape 3.7.0 software (Figure 2C) to clarify the main components of TY for treating RA. The network diagram contained 231 nodes and 511 edges (two herbs, 17 active ingredients, one disease, and 211 potential therapeutic targets). The size of each node represents the corresponding connectivity value. A larger area represents a greater connectivity value. Finally, the related topological analysis showed that kaempferol, quercetin, and octanoic acid were the core active components of TY against RA. Among them, the ADMET parameters and molecular structure description of its core active components kaempferol and quercetin have been widely studied.^24^, ^25^, ^26^, ^27^

#### 3.1.4 Analysis of the PPI network and obtaining core targets

**Figure 3:**
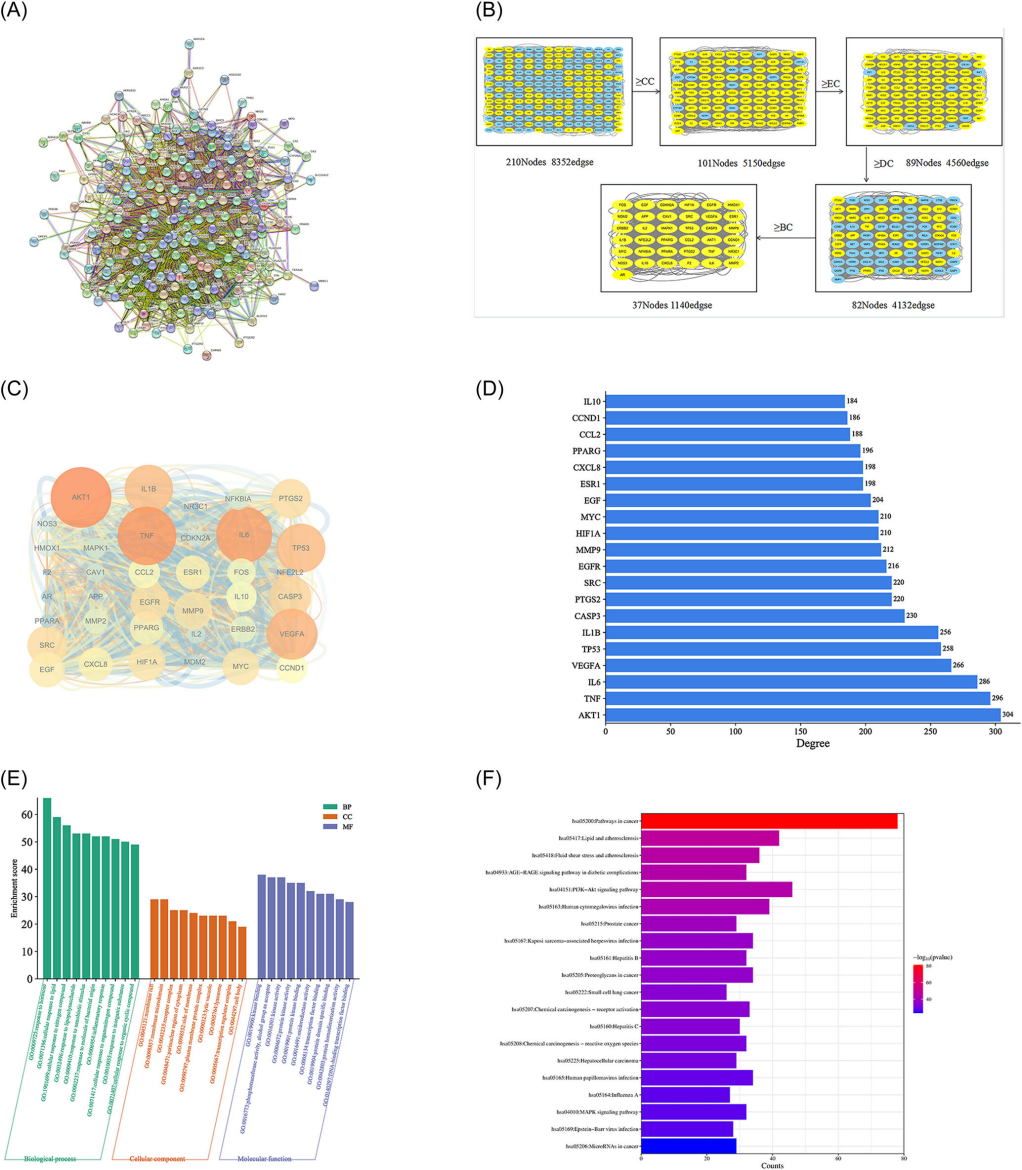
Screening the core targets and GO and KEGG pathway enrichment analyses. (A) The protein–protein interaction network of targets of *Rhodiola rosea*–*Euonymus alatus* drug pair (TY) in rheumatoid arthritis (RA). (B) Flowchart for screening core targets of TY in the treatment of RA. The yellow color was the target that met the screening conditions. (C) Network diagram of 37 core targets. The size and color depth of nodes are positively correlated with their degrees. (D) The bar chart of the top 20 core targets. The x‐axis represents the correlation degree and the y‐axis represents the core target. (E) The top 10 significantly enriched (*p* < .05) terms in biological process, cellular component, and molecular function of GO analysis were selected. The y‐axis represents the enrichment count of the target, and the x‐axis represents the GO category of the target gene. (F) The top 20 pathways with significantly enriched (*p* < .05) were selected. The y‐axis represents the main pathway, and the x‐axis represents the counts.

The PPI network for the potential targets of TY in RA was constructed using the STRING database. Then, the minimum required interaction score was set to 0.9 (the highest confidence level) and hid disconnected nodes in the network to draw the PPI network (Figure 3A). The network diagram contained 210 nodes, 4176 edges, and the average node degree was 39.6. According to the mean values higher than CC, EC, DC, and BC, the core targets of TY were screened. The screening process is shown in Figure 3B. Further topological analysis obtained 37 core targets (Figure 3C). Darker color and larger area of nodes indicated that the node was more important. Taking the abscissa as the correlation degree and the ordinate as the core target, the bar chart of the core target was drawn (Figure 3D). Ultimately, the top‐ranked targets, such as AKT1, TNF, IL‐6, vascular endothelial growth factor A (VEGFA), and IL‐1β, were found as the major targets of TY in the treatment of RA. As suggested by the results, TY improves RA through several potential targets.

#### 3.1.5 GO and KEGG pathway enrichment analyses

In total, 2538 GO terms were significantly enriched (*p* < .05), including 2172 biological process (BP) terms, 117 cellular component (CC) terms, and 249 molecular function (MF) terms. Based on the number of enriched genes, the top 10 significantly enriched terms in the BP, CC, and MF categories were displayed (Figure 3E). The results showed that the main BP terms were inflammatory response, response to hormones, and cellular response to lipopolysaccharide. The main CC terms were membrane raft, membrane microdomain, receptor complex, and perinuclear region of cytoplasm. The main MF terms were kinase binding, protein kinase binding, oxidoreductase activity, and transcription factor binding. It was demonstrated that TY ameliorates RA through several mechanisms.

Furthermore, 210 signaling pathways were significantly enriched (*p* < .05). Based on the number of enriched genes, the top 20 signaling pathways were selected for displaying (Figure 3F). The main pathways were PI3K/AKT signaling pathway and MAPK signaling pathway. These results suggest that TY can effectively prevent and treat RA through multiple targets and multiple pathways.

#### 3.1.6 Molecular docking analysis

**Figure 4:**
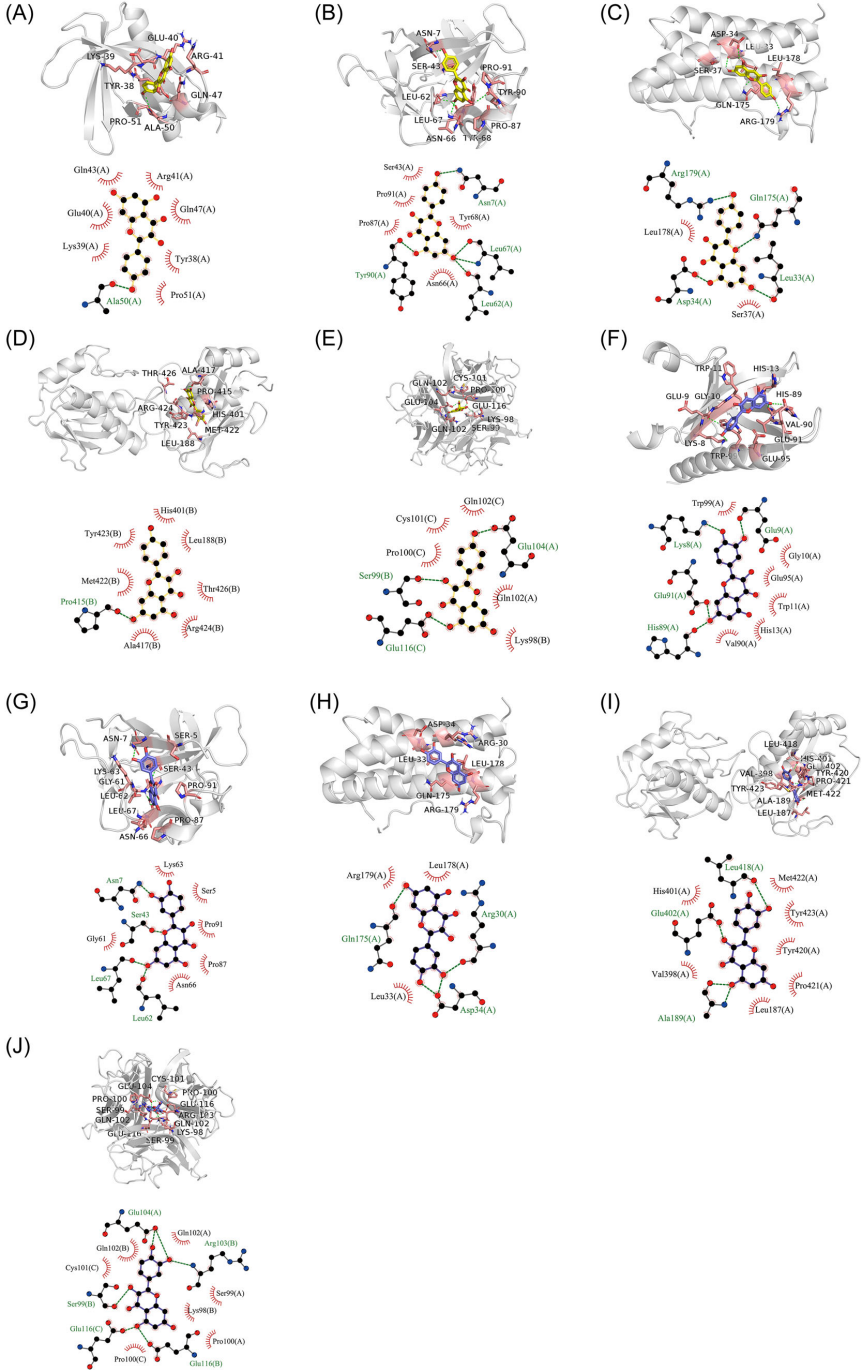
Visual molecular docking simulation of kaempferol and quercetin and core target AKT1, interleukin (IL)‐1β, IL‐6, matrix metalloproteinase (MMP)9, and tumor necrosis factor (TNF)‐α. (A) kaempferol‐AKT1; (B) kaempferol‐IL‐1β; (C) kaempferol‐IL‐6; (D) kaempferol‐MMP9; (E) kaempferol‐TNF‐α; (F) quercetin‐AKT1; (G) quercetin‐IL‐1β; (H) quercetin‐IL‐6; (I) quercetin‐MMP9; (J) quercetin‐TNF‐α.

The core targets, including IL‐1β, IL‐6, and TNF‐α, were docked with the active components, including kaempferol, octanoic acid, and quercetin. Docking score is shown in Table 3. Lower docking score indicates higher stability. The results showed that quercetin and kaempferol had good binding activity with IL‐1β, IL‐6, and TNF‐α, and had strong binding affinity to MMP9. In addition, molecular docking was applied to further verify the binding modes of kaempferol and quercetin with AKT1, IL‐1β, IL‐6, TNF‐α, and MMP9 (Figure 4).

**Table 3:** The docking score between main active components and core targets.

Target	PDB ID	Center (X, Y, Z)	Docking score/kcal·mol^−1^		
			(Kaempferol)	(Octanoic acid)	(Quercetin)
AKT1	1H10	21.612, 14.476, 9.985	−6.1	−4.3	−6.0
IL‐1β	1HIB	19.495, 2.994, 73.515	−7.2	−4.5	−7.1
IL‐6	1ALU	2.599, −20.016, 8.749	−6.6	−4.2	−7.1
TNF‐α	1A8M	20.083, 49.892, 39.738	−9.7	−5.2	−9.5
MMP9	1GKC	54.587, 21.248, 129.553	−8.6	−5.2	−8.5

Abbreviations: IL, interleukin; MMP, matrix metalloproteinase; TNF‐α, tumor necrosis factor α.

### 3.2 Results of in vitro study of TY on RA

#### 3.2.1 The effects of TY on the proliferation of HFLS‐RA cells

**Figure 5:**
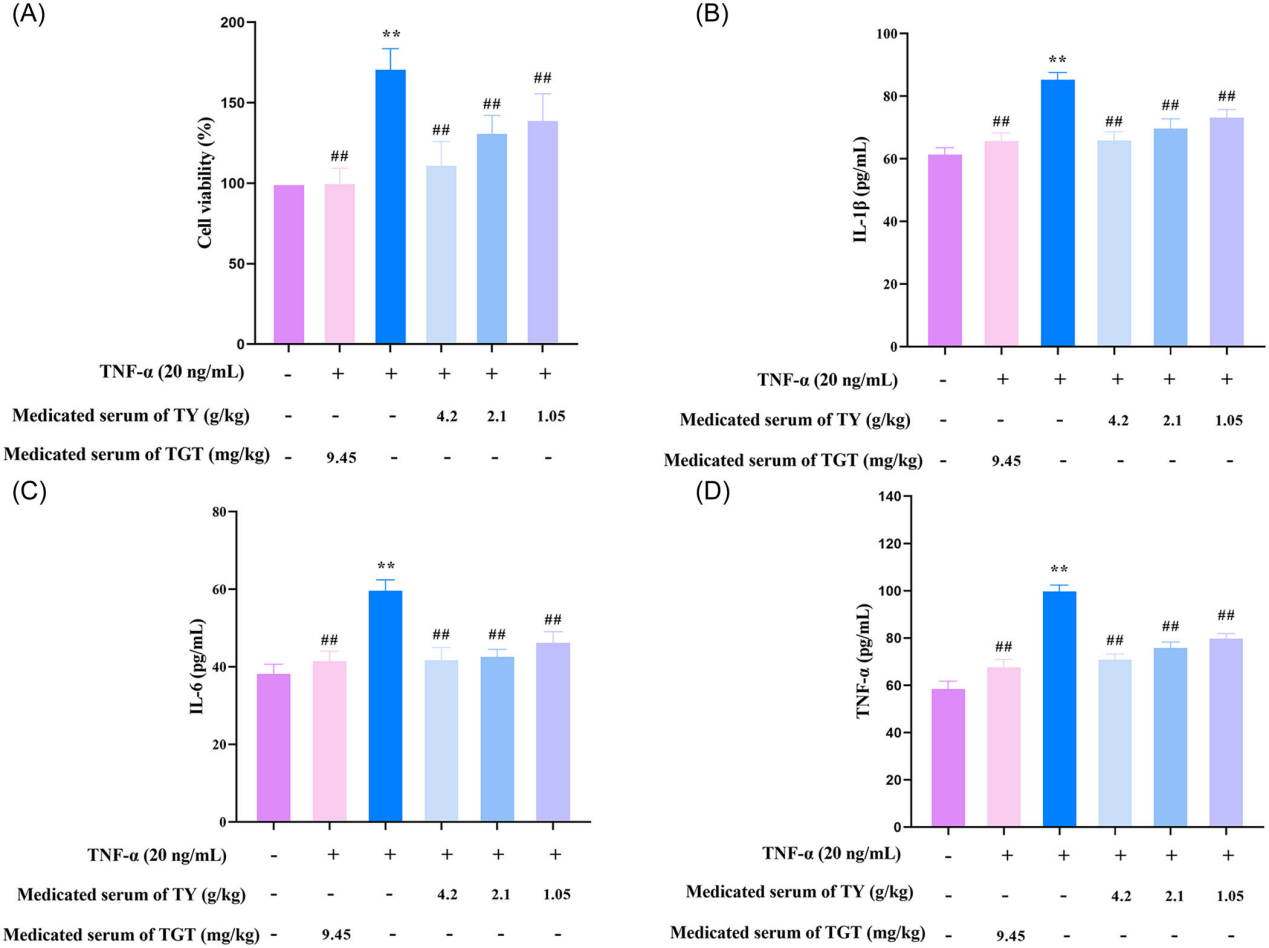
Effects of *Rhodiola rosea*–*Euonymus alatus* drug pair (TY) on tumor necrosis factor (TNF)‐α‐induced Fibroblast‐like synovial cells in human rheumatoid arthritis (HFLS‐RA) cells. (A) Effects of TY on HFLS‐RA cell viability. (B–D) The effect of TY on the production of inflammatory cytokines interleukin (IL)‐1β, IL‐6, and TNF‐α in HFLS‐RA cells. Data are presented as mean ± SD (*n*  =  6 in each group). **p < .01 versus control group; ^##^ *p* < .01 versus TNF‐α group.

Based on the results of network pharmacology, we investigated the effects of TY on synovial fibroblasts in vitro and explored the possible mechanism. We investigated the effects of TY on the proliferation of HFLS‐RA cells stimulated with TNF‐α. The TNF‐α group significantly increased proliferation (*p* < .01), compared with control group. Medicated serum of TY (1.05, 2.1, and 4.2 g/kg) dose‐dependently and TGT inhibited the proliferation of HFLS‐RA cells, compared with TNF‐α group (*p* < .01) (Figure 5A). The results indicated that medicated serum of TY (1.05, 2.1, and 4.2 g/kg) significantly suppressed the proliferation of HFLS‐RA cells after stimulation by TNF‐α.

#### 3.2.2 Effects of TY on the production of inflammatory cytokines in HFLS‐RA cells

Based on the results of ELISA (Figure 5B–D), the levels of IL‐6, IL‐1β, and TNF‐α significantly increased in the cell supernatants of the TNF‐α (20 ng/mL) group compared with the control group (*p* < .01). Drug‐containing serum of TY (1.05, 2.1, and 4.2 g/kg) and TGT significantly downregulated IL‐6, IL‐1β, and TNF‐α in the cell supernatants (*p* < .01). Therefore, TY significantly inhibited inflammatory cytokine production in HFLS‐RA cells.

### 3.3 Results of in vivo study of TY on RA

#### 3.3.1 Effect of TY on CIA rats

**Figure 6:**
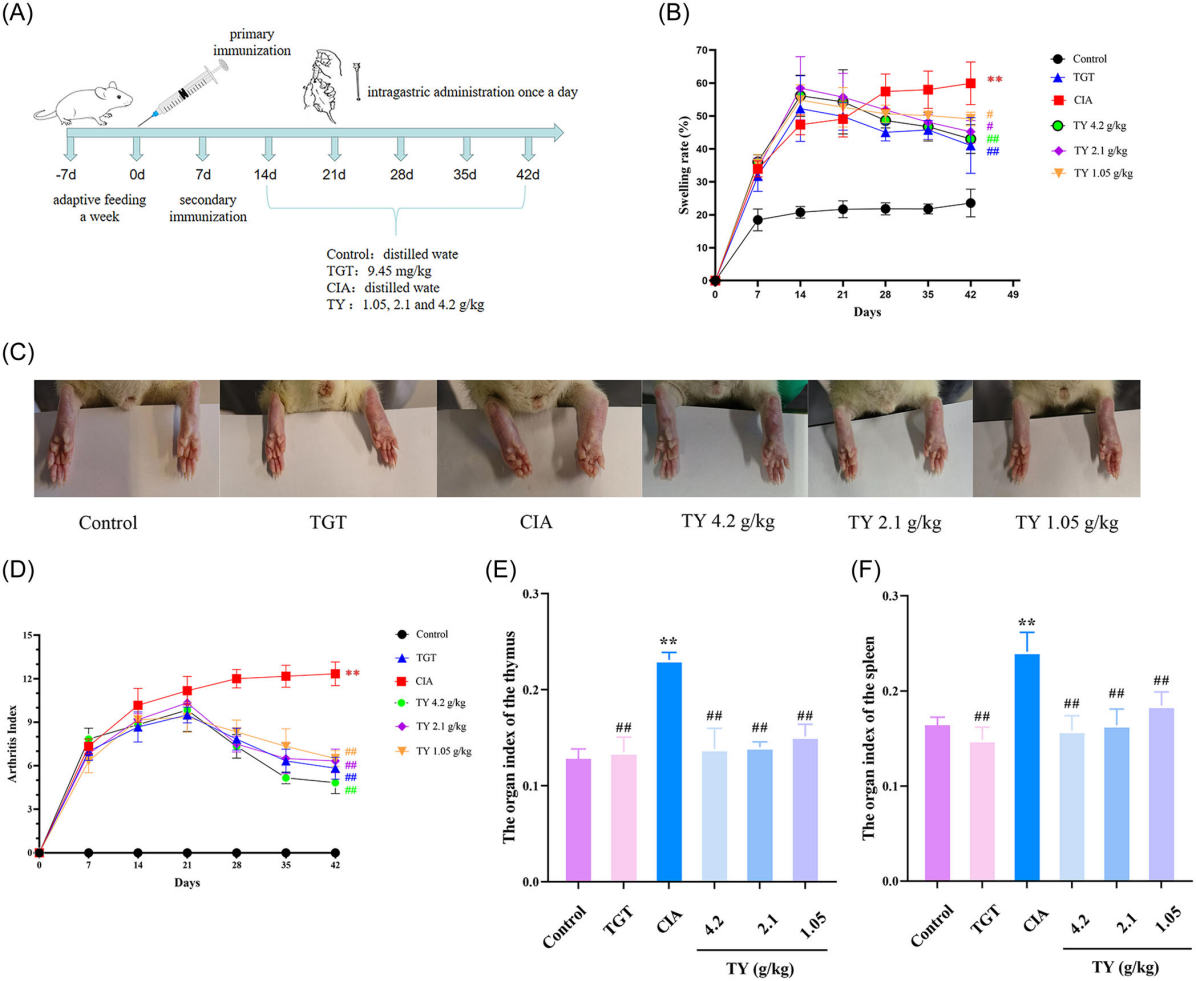
Therapeutic effects of *Rhodiola rosea*–*Euonymus alatus* drug pair (TY) in collagen‐induced arthritis (CIA) rats. (A) Diagram of CIA rat model induction and TY intervention. (B) Effects of TY on the swelling rate of CIA rats, respectively. (C) Representative photos of morphological changes of the left hind paw of different groups. (D) Arthritis scores were evaluated after intragastric administration of TY in CIA rats. (E and F) Organ index (g/100 g) of the spleen and thymus in each group. Data are presented as mean ± SD (*n* = 6 rats in each group). **p < .01 versus control group; ^##^ *p* < .01, ^#^ *p* < .05 versus CIA model group.

To further verify the efficacy and mechanisms of TY on RA. The rat model of CIA was reproduced (Figure 6A). The swelling rate of paws and arthritis scores were used to evaluate the effects of TY on CIA. Compared with the control group, the limbs of model and treated group rats were obviously swollen since 7th day, and the highest swelling rate was achieved on Day 14. According to the significant increase in swelling rate and AI score, CIA rats were successfully modeled. After 28 days of treatment with TY and TGT, joint swelling significantly improved (Figure 6B,C), and arthritis score significantly decreased (Figure 6D). We further monitored the organ index of the spleen and thymus. Compared with the control group, the organ index of the thymus and spleen significantly increased in the model group (Figure 6E,F), while TY and TGT effectively reduced the organ index of the thymus and spleen. The results showed that TY can improve arthritis in rats and has some immunomodulatory properties.

#### 3.3.2 Effect of TY on H&E staining of knee joint in CIA rats

**Figure 7:**
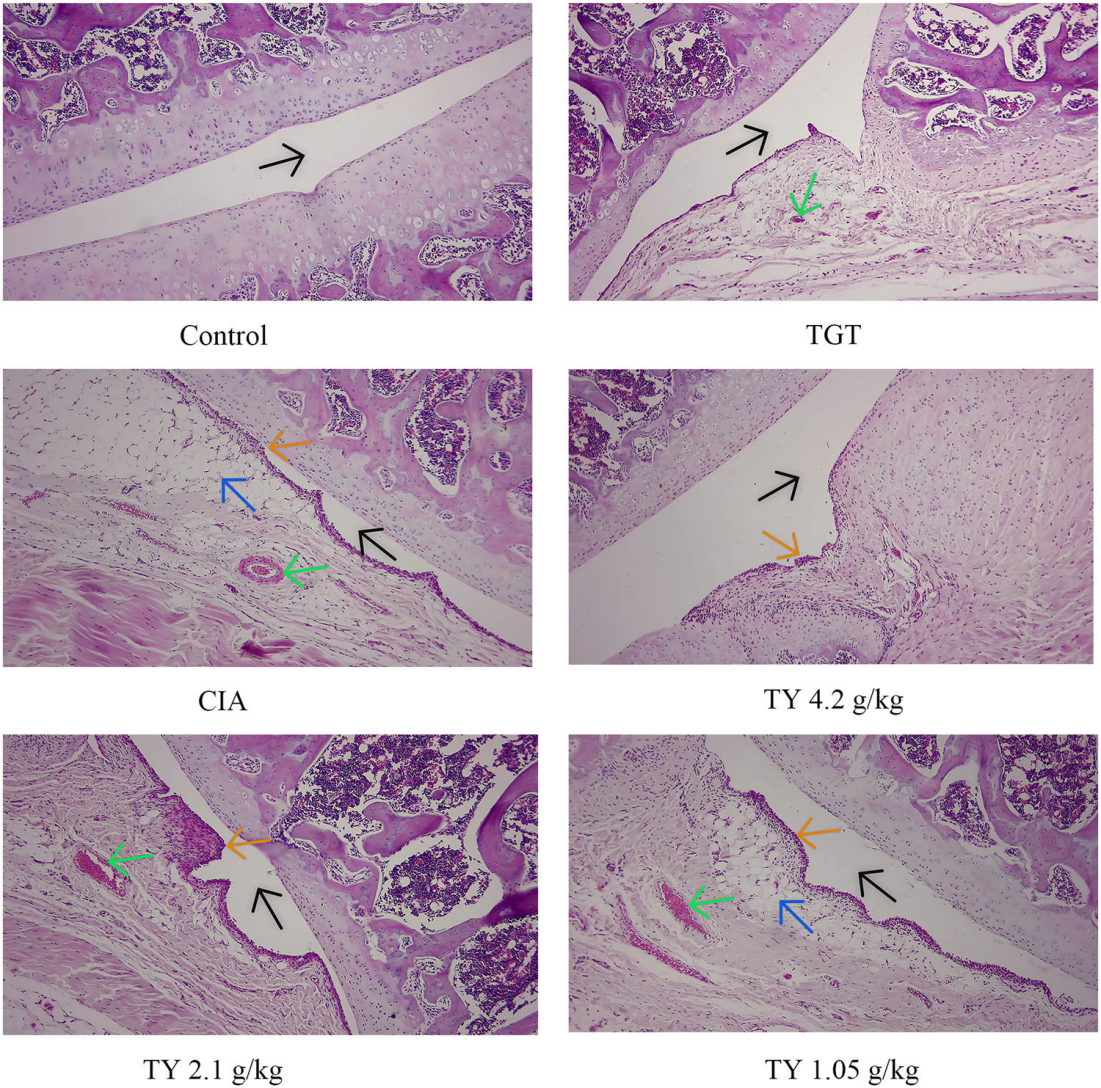
The synovium of the knee joint was sectioned for hematoxylin eosin staining. Representative joint tissue sections are shown (×10). Orange arrows: inflammatory cell infiltration; Black arrows: articular cavity; Green arrows: pannus; Blue arrows: fibroblast proliferation.

For examining the histopathological changes in the knee joints, H&E staining was performed. The knee joints of normal rats showed an intact articular cartilage structure with evenly distributed chondrocytes. However, rats in the modeling group exhibited marked pathological changes, such as thickened and expanded synovium with diffuse infiltration of inflammatory cells, significant proliferation of fibroblasts, and villous extension into the cartilage accompanied by pannus formation. Compared with the model group, treatment with TY and TGT markedly ameliorated inflammatory cell infiltration, diminished synovial hyperplasia, pannus formation, and fibroblasts proliferation (Figure 7). The results indicated that TY mitigated the histopathological alterations in the knee joints of CIA rats and reduced the severity of RA.

#### 3.3.3 Effects of TY on the inflammatory response in CIA rats

**Figure 8:**
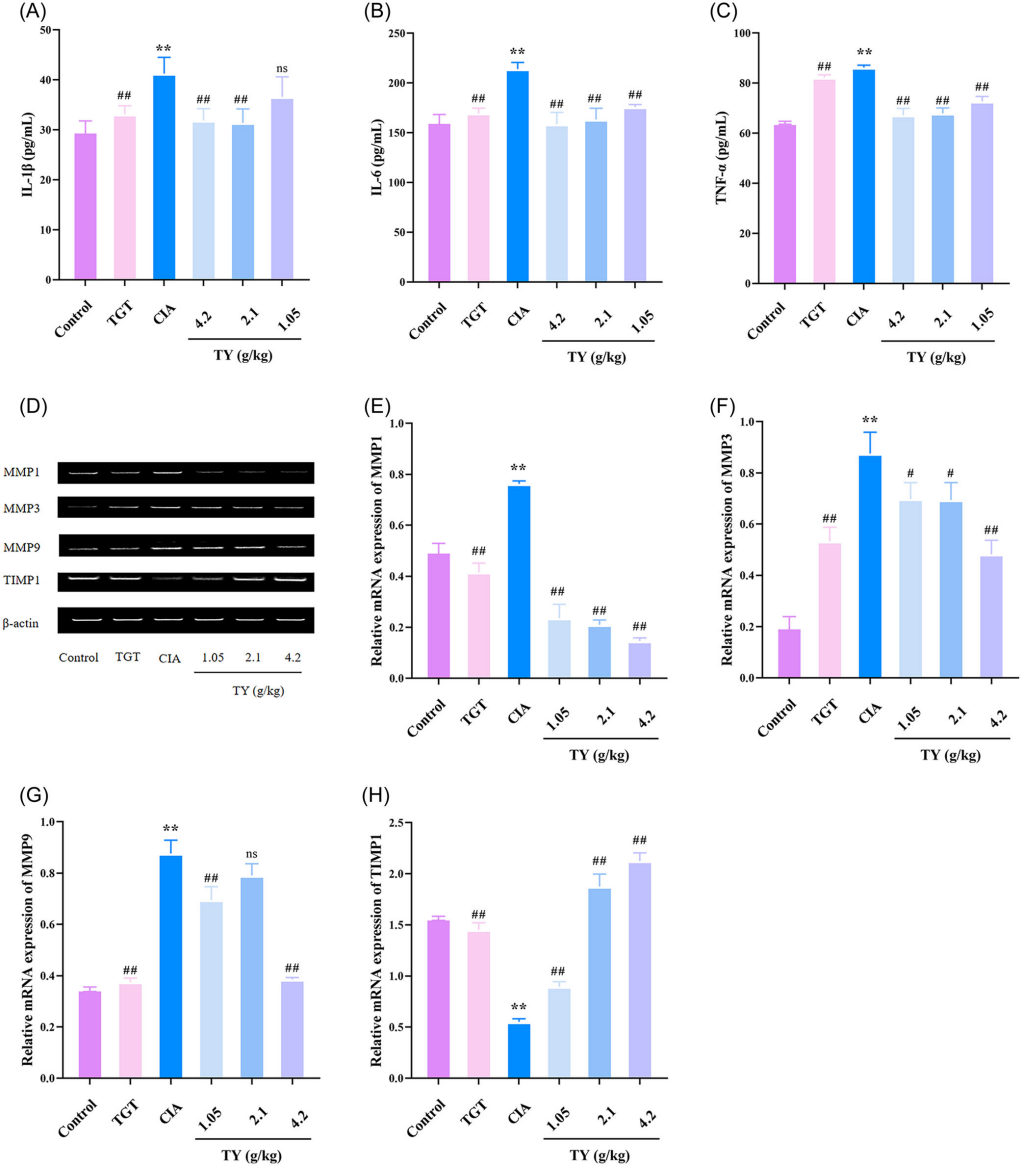
*Rhodiola rosea*–*Euonymus alatus* drug pair alleviated inflammation and inhibited angiogenesis in the synovial tissues of collagen‐induced arthritis (CIA) rats. (A–C) The serum levels of proinflammatory cytokines such as interleukin (IL)‐1β, IL‐6, and tumor necrosis factor‐α were measured by enzyme‐linked immunosorbent assay kit. (D–H) The mRNA levels of matrix metalloproteinase (MMP)1, MMP3, MMP9, and tissue inhibitors of MMPs 1 in synovial tissues were measured by quantitative real‐time polymerase chain reaction assays. Data are presented as mean ± SD (*n*  =  3 in each group). **p < .01 versus control group; ^##^ *p* < .01, ^#^ *p* < .05 versus CIA model group; ns no statistical significance.

In‐vitro experiments indicated that TY decreased inflammatory reactions in HFLS‐RA cells. Thus, we examined the anti‐inflammatory properties of TY in vivo. Compared with the control group, the serum levels of IL‐1β, IL‐6, and TNF‐α significantly increased in the model group (*p* < .01) (Figure 8A–C). Interestingly, TY and TGT significantly inhibited the serum levels of IL‐1β, IL‐6, and TNF‐α, compared with the model group (*p* < .01). The results showed that TY suppressed the inflammatory response in rats.

#### 3.3.4 Effects of TY on MMP1, MMP3, MMP9, and TIMP1 mRNA in CIA rats

To investigate the molecular mechanisms by which TY improves RA, the mRNA levels of MMP1, MMP3, MMP9, and TIMP1 were detected by RT‐qPCR. The mRNA levels of MMP1, MMP3, and MMP9 were significantly increased and the mRNA level of TIMP1 was decreased in the model group compared with the control group (*p* < .01). Compared with the model group, TY and TGT significantly decreased the mRNA levels of MMP1, MMP3, and MMP9 and increased the mRNA levels of TIMP1 (*p* < .05 and *p* < .01) (Figure 8D–H).

#### 3.3.5 Effects of TY on PI3K, p‐AKT, MMP1, MMP3, MMP9, and TIMP1 proteins in CIA rats

**Figure 9:**
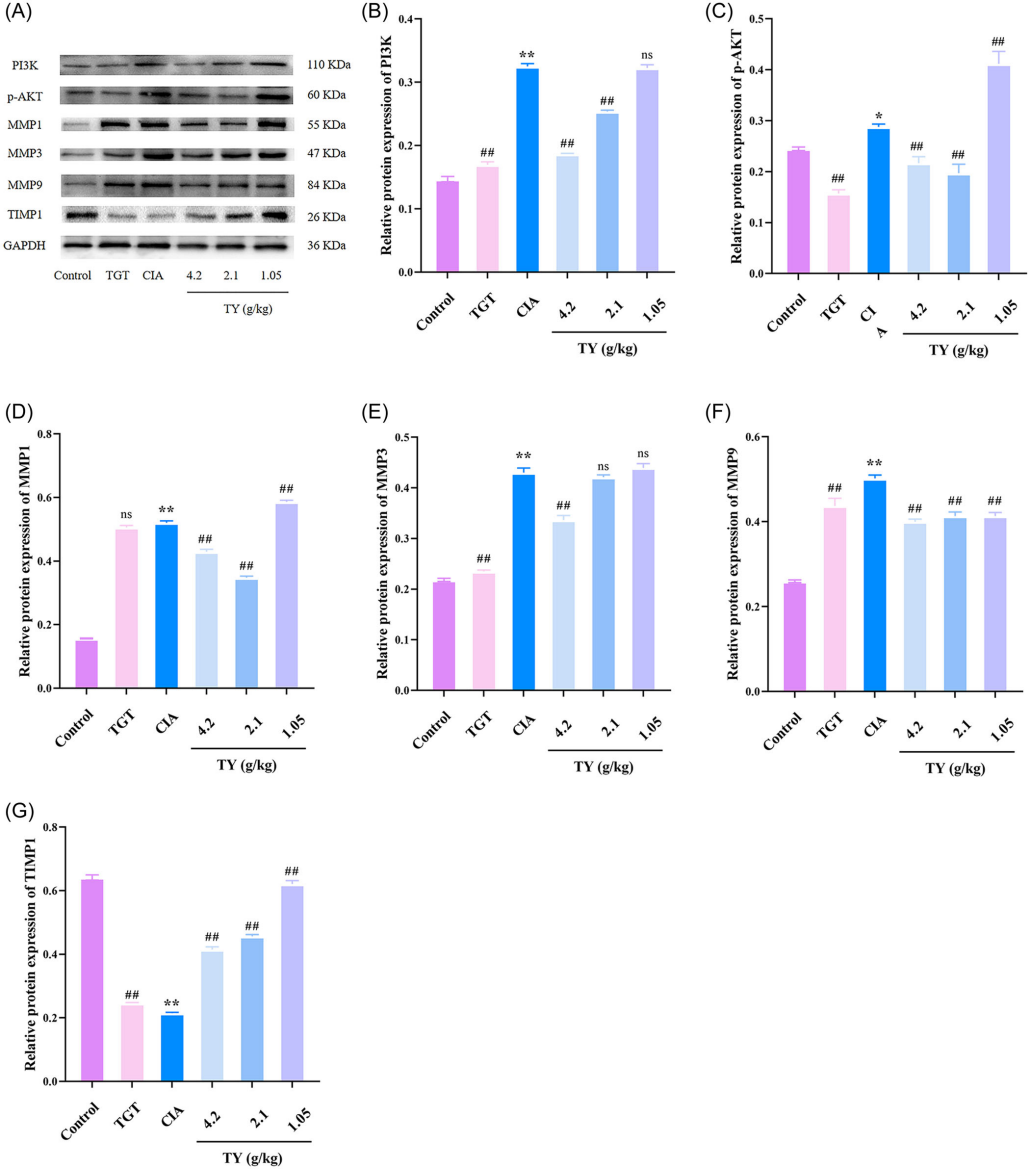
The effects of *Rhodiola rosea*–*Euonymus alatus* drug pair on phosphatidylinositide 3‐kinases (PI3K)/AKT pathway and matrix metalloproteinase/tissue inhibitors of MMPs (TIMP) balance in vivo. (A) Western blot analysis showing PI3K, p‐AKT, MMP1, MMP3, MMP9, and TIMP1 levels. (B and C) The protein levels of PI3K and p‐AKT in synovial tissues were measured by Western blot analysis. (D–‐G) The protein levels of MMP1, MMP3, MMP9, and TIMP1 in synovial tissues were measured. Data are presented as mean ± SD (*n*  =  3 in each group). **p < .01, *p < .05 versus control group; ^##^ *p* < .01, versus collagen‐induced arthritis model group; ns no statistical significance.

Western blot analysis was performed to further investigate the specific anti‐inflammatory mechanisms (Figure 9A–G). The expression levels of PI3K, p‐AKT, MMP1, MMP3, and MMP9 were upregulated and the expression level of TIMP1 was downregulated compared with the control group (*p* < .01). TY and TGT significantly decreased the protein levels of PI3K, p‐AKT, MMP1, MMP3, and MMP9, and increased the protein levels of TIMP1 compared with the model group (*p* < .05 and *p* < .01). These findings confirmed that TY inhibited the PI3K/ATK signaling pathway, downregulated IL‐1β, IL‐6, and TNF‐α, lowered the expression of angiogenic factors such as MMP1, MMP3, and MMP9, and upregulated the expression of TIMP1, as an angiogenesis inhibitor.

## 4 DISCUSSION

RA is a “Bi syndrome” in traditional Chinese medicine. The pathogenic mechanism is asthenia in origin and asthenia in superficiality. The pathogenesis of RA is not yet clear and is influenced by many factors, including genetic and environmental factors and immune dysregulation. Experimental studies revealed that *R. rosea* can regulate the expression of anti‐inflammatory/proinflammatory factors through the transcription factors Foxp3/Stat3/ROR‐γt, thereby inhibiting the abnormal proliferation of HFLS‐RA cells.^28^ The blood‐breaking and opening of the meridians effect of *E. alatus* can alleviate RA caused by blocked meridians and poor flow of Qi and blood.^29^ Clinical studies have also found that *R. rosea* can significantly improve the number of swollen joints, clinical symptoms and signs of RA, suggesting that *R. rosea* can significantly improve RA and Fuzheng Guben.^30^ The combination of *E. alatus* and Shanhaitang can effectively suppress inflammation and pain. It significantly improved joint pain, morning stiffness, swelling, erythrocyte sedimentation rate, rheumatoid factor, and other indicators.^31^ Various pharmacological studies confirmed that components of *R. rosea* and *E. alatus* can effectively treat RA.^32^, ^33^, ^34^ As this study was the first to use *R. rosea* in combination with *E. alatus*, it is difficult to fully elucidate its potential active compounds and precise pharmacological mechanisms in treating RA.

In this study, we conducted a systemic study using the combination of network pharmacology and in vivo and in vitro experiments to elucidate the bioactive components and therapeutic mechanisms of TY on RA. First, 18 active components and 326 potential targets were screened by network pharmacology, including 210 main pathways and 2538 BPs. According to the “herb‐component‐target‐disease” network diagram, the main active components of TY against RA were quercetin, kaempferol, and octanoic acid. According to the enrichment analysis of the PPI network and KEGG pathway, TY ameliorates RA by regulating PI3K/AKT, MAPK and other signaling pathways through key targets such as AKT1, TNF, IL‐6, VEGFA, and IL‐1β. Through molecular docking, it was verified that kaempferol and quercetin, the main active components of TY, vigorously bind to AKT1, IL‐1β, IL‐6, MMP9, and TNF‐α.

**Figure 10:**
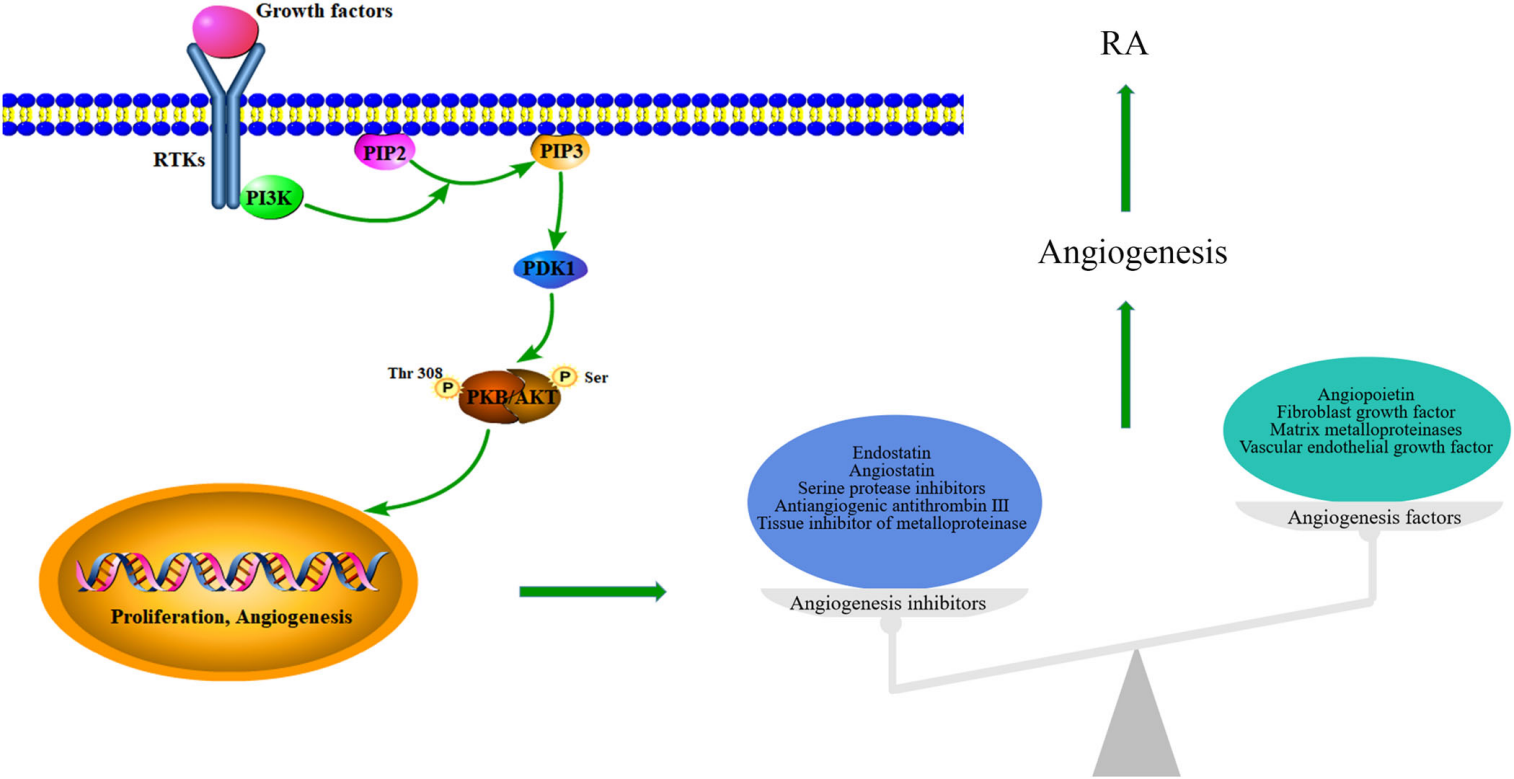
The mechanism by which angiogenesis is involved in RA. AKT/PKB, protein kinase B; PDK1, pyruvate dehydrogenase kinase 1; PI3K, phosphatidylinositide 3‐kinases; PIP2, phosphatidylinositol (4,5) bisphosphate; PIP3, phosphatidylinositol (3,4,5) triple‐phosphate; RA, rheumatoid arthritis; RTKs, receptor tyrosine kinases.

To further explore the mechanism by which TY ameliorates RA, we verified the core targets predicted by network pharmacology, including PI3K, AKT1, TNF‐α, IL‐1β, IL‐6, MMPs, and TIMP1. The PI3K/AKT signaling pathway links the proliferation of FLS to the inflammatory response. It transduces the signal from various growth factors and cytokines.^35^ It has been found that the PI3K/AKT signaling pathway is overactivated in FLS in RA.^36^ PI3K is a heterodimer lipid kinase and a key regulator of intracellular signals. AKT is a protein kinase that regulates cell survival and apoptosis. It is also a key downstream target of PI3K. PI3K binds to AKT and phosphorylates it, which triggers a series of reactions and promotes cell growth, proliferation, migration, invasion, and angiogenesis.^37^ MMPs are a type of zinc proteases that degrade the extracellular matrix and promote angiogenesis and pannus formation, aggravating joint pain, swelling, bone erosion, and cartilage destruction.^38^ The TIMP inhibit the proteolytic activity of MMPs. TIMPs can inhibit angiogenesis, cell proliferation, and apoptosis.^39^ MMPs and TIMPs play key roles in the self‐regulation of inflammation in RA.^40^, ^41^ Studies have shown that MMPs, such as MMP1, MMP3, and MMP9, are downstream targets of the PI3K/AKT signaling pathway and lead to cartilage degradation and joint damage. They are also closely related to the occurrence and development of RA.^42^ Relevant studies have found that activation of PI3K/AKT signaling pathway can dysregulate MMPS and TIMP expression and promote angiogenesis and pannus formation.^43^ IL‐1β, IL‐6, and TNF‐α are major proinflammatory cytokines. Studies have shown that proinflammatory cytokines such as TNF‐α, IL‐1β, and IL‐6 are deeply involved in the pathogenesis of RA. High expression of IL‐1β or TNF‐α activates the expression of IL‐6, which induces an inflammatory response, leading to bone destruction and pannus formation.^44^ Inflammatory cytokines such as TNF‐α, IL‐1β, and IL‐6 can activate MMPs, promote MMPs release, and upregulate their expression in HFLs‐RA.^45^, ^46^ Therefore, reducing the expression of IL‐1β, IL‐6, TNF‐α, and MMPs by inhibiting PI3K/AKT signaling pathway can markedly improve RA. The role of angiogenesis in the pathogenesis of RA is shown in Figure 10.

To further verify our findings, we conducted in vitro and in vivo experiments. In vivo, compared with the control group, the expression of PI3K, p‐AKT, IL‐1β, IL‐6, TNF‐α, MMP1, MMP3, and MMP9 significantly increased and the expression of TIMP1 decreased in the model group, indicating that the PI3K/AKT pathway is involved in RA, and controls the balance between MMPS and TIMP. Compared with the model group, the expression of PI3K, p‐AKT, IL‐1β, IL‐6, and TNF‐α decreased with different doses of TY, thereby inhibiting the expression of MMPs and increasing the expression of TIMP1. In‐vitro experiments showed the same results.

## 5 CONCLUSION

In summary, this study was the first study that used network pharmacology approaches and in vivo and in vitro experiments to comprehensively analyze the main compounds, targets, and pathways of TY in treating RA. We demonstrated that TY can inhibit HFLs‐RA cell proliferation, improve the symptoms of RA in rats, and inhibit the inflammatory response. The underlying mechanism was associated with the inhibition of the PI3K/AKT signaling pathway, regulating the balance between MMPS and TIMP, and inhibiting angiogenesis. This study provides a theoretical basis for the application of TY for treating RA in clinical settings.

This study has several limitations. First, only the top‐ranked core targets were selected for verification, and the correlation between their pathways requires further investigation. Second, at present, CIA rat models can well simulate the pathological characteristics of human RA, but there are species and endogenous differences between animal models and human diseases. So, further experimental validations are necessary to explore the mechanisms and support the clinical application of TY.

## AUTHOR CONTRIBUTIONS

**Qiu‐han Zheng**: Investigation; data curation; formal analysis; writing—original draft; writing—review and editing. **Lian‐yun Du**: Revised manuscript; writing—review and editing. **Ying Zhao**: Revised the manuscript. **Zhong Zhang**: Collected synovial tissue. **Song‐lan Piao**: Supervised experiment. **Zhi Pan**: Conceptualization; supervised study; funding acquisition; project administration. **Ying‐hang Wang**: Conceptualization; supervised study; funding acquisition; project administration. All authors studied and approved the manuscript.

## CONFLICT OF INTEREST STATEMENT

The authors declare no conflict of interest.

## ETHICS STATEMENT

This study involving human data from public databases GeneCards, TTD, DrugBank, and DisGeNET. Due to GeneCards, TTD, DrugBank, and DisGeNET belong to public databases and users can download relevant data for free for research and publish relevant articles. This study did not involve human experiments; the Animal Experimental Ethical Inspection From of Changchun University of Chinese Medicine confirms that this study would have had the need for ethics approval waived. As the in vitro experiments were performed on cell lines, approval from the ethics committee was not required for this study. All animal experiments were approved by the Experimental Animal Administration Committee of the Changchun University of Chinese Medicine (Approval No. 2021361) and conducted in accordance with institutional guidelines.

## Data Availability

The original contributions presented in the study are included in the article/Supplementary Material. Further data can be obtained from the corresponding authors upon request.
